# Evaluation of the anti-*Listeria* potentials of some plant-derived triterpenes

**DOI:** 10.1186/s12941-014-0037-1

**Published:** 2014-07-23

**Authors:** Dambudzo Penduka, Rebamang Mosa, Mthokozisi Simelane, Albert Basson, Anthony Okoh, Andy Opoku

**Affiliations:** 1 Department of Biochemistry and Microbiology, University of Zululand, P Bag X1001, KwaDlangezwa, 3886, South Africa; 2 Department of Biochemistry and Microbiology, University of Fort Hare, P. Bag X1314, Alice 5700, South Africa

**Keywords:** 3β-hydroxylanosta-9,24-dien-21-oic acid, Methyl-3β-hydroxylanosta-9,24-dien-21-oate, 3β-acetylursolic acid, Listeria, Protorhus longifolia, Mimusops caffra

## Abstract

**Background:**

Listeriosis is a fatal disease caused by pathogenic *Listeria* bacteria and it is most prevalent in immune-compromised individuals. The increase in numbers of immune-compromised individuals against a background of *Listeria* antibiotic resistance, limits listeriosis treatment options. This therefore calls for research into substitute treatments, of which, medicinal plants derived compounds offer a viable alternative.

**Methods:**

The broth microdilution assay was used to determine the minimum inhibitory concentration (MIC) and minimum bactericidal concentration (MBC) of three plant triterpenes namely 3β-hydroxylanosta-9,24-dien-21-oic acid, methyl-3β-hydroxylanosta-9,24-dien-21-oate and 3β-acetylursolic acid, against *Listeria monocytogenes*, *Listeria ivanovii* and *Listeria grayi* species. The chequerboard method was used to assess the interactions between the triterpenes and conventional antibiotics: ampicillin, neomycin, gentamicin and penicillin G. The lactate dehydrogenase membrane damage method was used to assess the triterpenes’ membrane damaging potentials against the *Listeria* bacteria.

**Results:**

The triterpenes’ MIC values were found to range from 0.185 to 1.67 mg/ml while, the MBC determination assay results revealed that the test triterpenes were bacteriostatic against the *Listeria* bacteria. The interactions involving 3β-hydroxylanosta-9,24-dien-21-oic acid were mainly additive with ampicillin and synergistic with neomycin, gentamicin and penicillin G. The interactions involving methyl-3β-hydroxylanosta-9,24-dien-21-oate were mainly antagonistic with ampicillin, indifferent with neomycin, ranging from synergistic to indifference with gentamicin and synergistic with penicillin G. The interactions involving 3β-acetylursolic acid were mainly indifferent with ampicillin, synergistic with neomycin and gentamicin while ranging between synergistic and additive with penicillin G. The low levels of cytosolic lactate dehydrogenase released from the cells treated with 4× MIC concentration of the triterpenes in comparison to that of cells treated with 3% Triton X-100 proved that membrane damage was not the mode of action of the triterpenes.

**Conclusion:**

This study therefore shows the potential that these plant triterpenes have in listeriosis chemotherapy especially as shown by the favourable interactions they had with penicillin G, one of the antibiotics of choice in listeriosis treatment.

## Introduction

There are currently fifteen known *Listeria* species [[1]], but only two of the species are known to be pathogenic; *L. monocytogenes* is pathogenic to humans and animals while *L. ivanovii* is pathogenic to animals only. *L. monocytogenes* causes the human fatal disease listeriosis with a case fatality rate of 20-30%; the disease can be in either of two forms, gastrointestinal non-invasive listeriosis which is usually self-limiting or the invasive listeriosis which can be fatal [[2]].

Contaminated foods such as raw vegetables, meats and ready to eat foods are the major source of pathogenic *Listeria*, such that the gastrointestinal tract is the bacteria’s primary site of entry [[3]]. The bacteria then colonises the intestine leading to intestinal translocation and at this stage the listeriosis is non-invasive however, if the immune system does not control the infection, the listeriosis progresses to become invasive as the pathogen disseminates to the bloodstream and mesenteric lymph nodes [[2],[4]]. The bacterium may then replicate in the liver and spleen, such that control of the bacterium at this point is dependent upon T cell-mediated immunity. However, in immune-compromised individuals the *Listeria* spreads to the central nervous system or in the case of pregnant women it crosses the placental barrier resulting in infection of the foetus [[2],[4],[5]].

The pathogenic *Listeria* has an intracellular life cycle that includes invading host cells by using adhesion proteins internalin A and internalin B to bind to the host-cell membrane receptors E-cadherin and Met, replication in the cytoplasm after phagosomal escape and cell to cell spread through ActA surface proteins’ polymerization of actin [[6],[7]].

Ampicillin and penicillin G are the first line drugs of choice for listeriosis treatment, with them being used in combination with an aminoglycoside mostly gentamicin in high risk patients (neonates aged less than 1 month, the elderly, immune-compromised individuals, pregnant women). Meropenem may be used in patients with mild allergies to penicillin, while sulfamethoxazole in combination with trimethoprim or vancomycin in combination with teicoplanin can be used in patients with severe allergies [[7],[8]].

The administration of appropriate and effective antibiotic therapy in high-risk patients to prevent invasive listeriosis is imperative [[7]]. A number of factors have however, limited listeriosis treatment options. The factors include; the characteristic life cycle of pathogenic *Listeria* to multiply intracellularly and spread from cell-to-cell without leaving the protective environment of the host’s cells [[4]]; the limited treatment options due to the negative side effects of some of the antibiotics especially in pregnant women, children, patients with allergies and in organ transplant recipients [[7]]; the high mortality rate even despite early antibiotic treatment [[8]] and the reports on antibiotic resistance among *Listeria* species [[9]]. These factors therefore, show the need for a continuous search for newer and more effective listeriosis treatment options.

Medicinal plants have been used since time immemorial to treat various types of illnesses and most have formed the basis of some effective antimicrobial agents [[10]]. In this connection this study focused on the anti-*Listerial* activities of three triterpenes isolated from two traditional medicinal plants namely *Protorhus longifolia* and *Mimusops caffra*. Plant triterpenes comprise a diverse chemical group of active principles and have been reported to possess anti-inflammatory, antiviral, antimicrobial, and antitumor activities [[11]].

*Protorhus longifolia* is an evergreen plant that grows up to a height of 18 m with a trunk diameter of about 1 m and belongs to the tropical and sub-tropical family of Anacardiaceae [[12]]. The genus *Protorhus* Engl is mostly found in Madagascar and only two species are found in Africa namely *Protorhus namaquensis* (Namibian/South African border) and *Protorhus longifolia* found in southern parts of Africa, and mostly abundant in the sub-tropical forests of KwaZulu Natal in South Africa [[12]]. The tea from the mixture of the barks of *Protorhus longifolia* and *Hippobromus pauciflorus* is known to treat heartwater and diarrhea in cows [[13]]. Its leaf extracts have been shown to possess both antibacterial and antifungal activities *in-vitro* [[14]] and Mosa *et al*. [[15]] isolated two triterpenes from the chloroform extract of the bark namely 3-oxo-5α-lanosta-8,24-dien-21-oic acid and 3β-hydroxylanosta-9,24-dien-21-oic acid, which exhibited anti-platelet aggregation activities.

*Mimusops caffra* belongs to the Sapotaceae family and the genus *Mimusops* consists of 30 species, some of which grow in tropical and sub-tropical regions of Asia [[16]]. *Mimusops caffra* can be found in Southern Africa especially in Mozambique and in the Kwazulu Natal and Eastern Cape provinces of South Africa [[17]]. Its traditional medicinal properties include wounds and sore healing [[17]]. Ursolic acid isolated from the leaves of the plant, was shown to exhibit anti-*plasmodial* activities against *Plasmodium falciparum* (D10) [[17]].

Herein, this study provides a scientific basis for the anti-*Listeria* activities of 3β-hydroxylanosta-9,24-dien-21-oic acid and methyl-3β-hydroxylanosta-9,24-dien-21-oate isolated from *Protorhus longifolia* and 3β-acetylursolic acid a derivative of ursolic acid which was isolated from *Mimusops caffra in-vitro*.

## Method

### Test triterpenes

**Figure 1:**
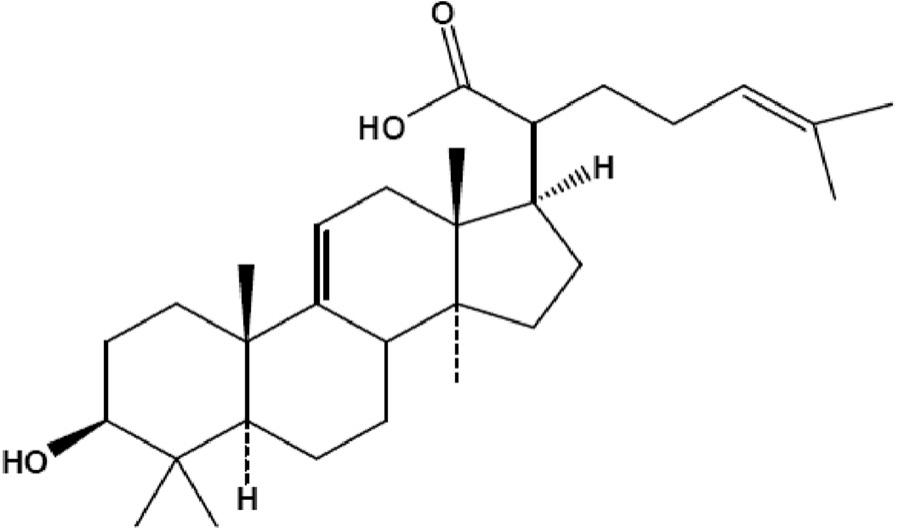
**Chemical structure of 3β-Hydroxylanosta-9,24-dien-21-oic acid** [[15]]**.**

**Figure 2:**
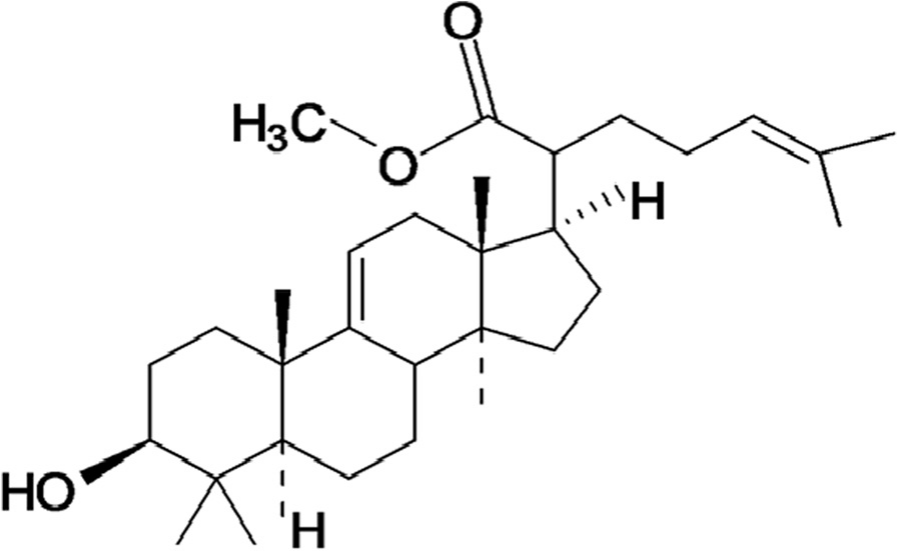
**Chemical structure of methyl-3β-hydroxylanosta-9,24-dien-21-oate** [[18]]**.**

**Figure 3:**
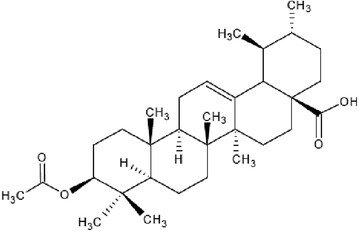
**Chemical structure of 3β-acetylursolic acid** [[17]]**.**

The 3β-hydroxylanosta-9,24-dien-21-oic acid and methyl-3β-hydroxylanosta-9,24-dien-21-oate were previously isolated and characterized from the stem bark of *Protorhus longifolia* by Mosa *et al*. [[15]] and Mosa [[18]] respectively. The 3β-acetylursolic acid was previously derivatised from ursolic acid isolated from the leaves of *Mimusops caffra* by Simelane *et al*. [[17]]. The chemical structures of the triterpenes are as shown in Figures 1, 2 and 3.

#### Test antibiotics

The antibiotics penicillin G, ampicillin, neomycin and gentamicin obtained from Sigma (South Africa) were used. These were dissolved in sterile distilled water to make the test concentration.

#### Test *Listeria*

The test *Listeria* was obtained from the culture collection of the Applied and Environmental Microbiology Research Group (AEMREG) at the University of Fort Hare, Alice, South Africa. The organisms included a referenced *Listeria monocytogenes* ATCC- 19115 strain as well as environmental isolates of *Listeria grayi* (LAL 3) and *Listeria ivanovii* (LDB 11) both previously isolated from waste water effluents by Odjadjare *et al*. [[19]].

#### MIC determination

The MIC’s of the triterpenes were determined according to the microbroth dilution method of EUCAST [[20]] as described by Penduka and Okoh [[21]] in 96 well microtiter plates. The test *Listeria* was standardised to match the 0.5 McFarland standard. A starting concentration of 5 mg/ml of each triterpene dissolved in 10% DMSO was serially diluted double fold in double strength Mueller-Hinton Broth to make different test concentrations of the compounds in the wells. A volume of 20 μl of the test organisms was introduced to 100 μl of the test triterpene in broth. The antibiotics penicillin G, ampicillin, neomycin and gentamicin were used as positive controls. The 10% DMSO was also tested for its possible anti-*Listeria* activities. Sterility wells were also included and the whole assay was perfomed in triplicates. The plates were then incubated at 37°C for 18-24 h, after which results were read visually by adding 40 μl of 0.2 mg/ml of ρ-iodonitrotetrazolium violet (INT) into each well. A colour change (viewing with the naked eye) from colourless to purple, indicated actively growing bacteria based on the oxidation-reduction reaction in which electrons are transferred from NADH (a product of the oxidation of threonine to 2-amino-3-ketobutyrate) to INT which then forms the red formazan which is purple in colour. The MIC was recorded as the lowest concentration of the triterpene or antibiotic that prevented the growth of the organism after 18-24 h.

#### MBC determination

The MBC was determined using the method described by Sudjana *et al*. [[22]] with some minor modifications. Briefly, the test triterpenes and antibiotics were serially diluted in double strength Mueller-Hinton broth in 96 well microtitre plates to make different test concentrations starting with 8× MIC value of the test antibacterial agent up to its MIC value against each organism. The test *Listeria* was standardised to match the 0.5 McFarland standard and 20 μl of the organism was inoculated into each well containing 100 μl of the test antibacterial agent in broth. The plates were incubated for 18-24 h after which 15 μl was subcultured from each well and inoculated onto fresh Mueller-Hinton agar plates. The agar plates were then incubated for 18-24 h after which the MBC was interpreted as the minimum concentration of the antibacterial agent that prevented the growth of viable colonies on the Mueller-Hinton agar after incubation.

#### Interactions

The interactions between the triterpenes and selected antibiotics were perfomed in 96 well microtiter plates as described by Penduka *et al*. [[23]] using the chequer-board method. The starting antimicrobial combination in double strength Mueller-Hinton broth was serially diluted to make different test concentrations in the microtiter plate, with each well containing 100 μl of the test antimicrobial combination. A volume of 20 μl of the standardised 0.5 MacFarland test bacteria was added into the test wells. Sterility wells containing the broth only and growth control wells containing the bacteria and broth only were also added in each microtitre plate. The MIC’s of the test combination were determined after 18-24 h of incubation at 37°C using the same INT method mentioned in the MIC determination. The interactions were interpreted through the use of Fractional Inhibitory Indices (FIC). The FIC index of a triterpene (FIC_T_) was calculated as the ratio of the MIC value of the triterpene in combination over the MIC value of the triterpene alone, and the FIC index of the antibiotic (FIC_A_) was calculated as the ratio of the MIC value of the antibiotic in combination over the MIC value of the antibiotic alone. The overall FIC index (ΣFIC) was calculated as the summation of the FIC_T_ and the FIC_A_. The interactions were interpreted as synergism when the ΣFIC index ≤ 0.5, additive when 0.5 < ΣFIC index ≤ 1, and indifference when 1 < ΣFIC index < 4 whilst antagonism was defined as when the ΣFIC index is ≥4. The test was perfomed in triplicates.

#### Cytosolic lactate dehydrogenase assay for membrane damage determination

The cytosolic lactate dehydrogenase assay was carried out according to the methodology described by Soyingbe *et al*. [[24]] with some modifications. Standardized test *Listeria* cultures matching 0.5 MacFarland standard were grown for 18-24 h in a concentration of 4× MIC value of each triterpene after which, the mixture was centrifuged (5 000 × g for 5 mins). An aliquot of 50 μl from the supernatant was incubated with 50 μl mixed reaction solutions of a lactate dehydrogenase (LDH) activity assay kit (Sigma Aldrich), at room temperature and incubated for 30 min. After which, the absorbance was measured at 492 nm using a 96 well microplate reader (BiotekELx 808). Cultures grown in 3% Triton X-100 were used as the positive control. The extent of membrane damage was calculated as (E-C)/(T-C) × 100, where E is the experimental absorbance of the cell cultures incubated with the test triterpenes, C is the control absorbance of the cell medium and T is the 3% Triton X-100 treated cells supernatant.

## Statistical analysis

The results were reported as mean values of triplicate experiments.

## Results

### MIC determination

The MIC values of the triterpenes and the antibiotics are as shown in Table 1. The triterpenes isolated from *P. longifolia* had an MIC value of 0.185 mg/ml against all the three test *Listeria* bacteria, while the 3β-acetylursolic acid had an MIC value of 1.67 mg/ml against all the three *Listeria* bacteria. The 10% DMSO was found not to exhibit anti-*Listeria* activities *in-vitro*.

**Table 1:** **MIC determination of the triterpenes against the test** ***Listeria*** **bacteria**

**Test antibacterial agent**	***L. monocytogenes***	***L. ivanovii***	***L. grayi***
3β-hydroxylanosta-9,24-dien-21-oic acid (mg/ml)	0.185	0.185	0.185
methyl-3β-hydroxylanosta-9,24-dienoate (mg/ml)	0.185	0.185	0.185
3β-acetylursolic acid (mg/ml)	1.67	1.67	1.67
ampicillin (μg/ml)	0.014	0.005	R
penicillin G (μg/ml)	0.079	0.079	R
neomycin (μg/ml)	0.019	0.157	1.25
gentamicin (μg/ml)	0.157	0.157	0.625
10% DMSO	NA	NA	NA

Note: R denotes resistance towards the test antibacterial agent at a maximum test concentration of 100 μg/ml and NA denotes not active.

### MBC determination

The results of the MBC determination are as shown in Table 2. All the triterpenes were bacteriostatic against the *Listeria* at a maximum concentration of 8× MIC value of the test triterpene. The antibiotics were also mostly bacteriostatic except for neomycin and gentamicin against *L. grayi* whereby the antibiotics were bactericidal.

**Table 2:** **MBC determination of the triterpenes against the test** ***Listeria*** **bacteria**

**Test antibacterial agent**	***L. monocytogenes***	***L.ivanovii***	***L.grayi***
3β-hydroxylanosta-9,24-dien-21-oic acid (mg/ml)	>1.48	>1.48	>1.48
methyl-3β-hydroxylanosta-9,24-dienoate (mg/ml)	>1.48	>1.48	>1.48
3β-acetylursolic acid (mg/ml)	>13.36	>13.36	>13.36
ampicillin (μg/ml)	>0.112	>0.04	R
penicillin G (μg/ml)	> 0.632	>0.632	R
neomycin (μg/ml)	>0.152	>1.26	10
gentamicin (μg/ml)	>1.26	>1.26	5

Note: R denotes resistance towards the test antibacterial agent at a maximum test concentration of 100 μg/ml.

### Interactions of the triterpenes and conventional antibiotics

The interactions of the triterpenes and conventional antibiotics are as shown in Tables 3, 4, and 5. Penicillin G had synergistic and additive interactions only, while neomycin and gentamicin had interactions ranging from synergistic to indifference and ampicillin had interactions ranging from additive to antagonistic with the test triterpenes.

**Table 3:** Interactions of 3β-hydroxylanosta-9,24-dien-21-oic acid and the different antibiotics

	***L. grayi***	***L. monocytogenes***	***L. ivanovii***
Ampicillin	ND	(1) Additive	(1) Additive
Penicillin G	ND	(0.498) Synergy	(0.498) Synergy
Neomycin	(0.375) Synergy	(0.75) Additive	(0.497) Synergy
Gentamicin	(0.245) Synergy	(0.314) Synergy	(1.25) Indifference

Note: ND denotes not determined and (number) denotes the ΣFIC index value.

**Table 4:** Interactions of methyl-3β-hydroxylanosta-9,24-dien-21-oate and the different antibiotics

	***L. grayi***	***L. monocytogenes***	***L. ivanovii***
Ampicillin	ND	(4) Antagonistic	(4) Antagonistic
Penicillin G	ND	(0.498) Synergy	(0.498) Synergy
Neomycin	(1.5) Indifference	(3.05) Indifference	(1.01) Indifference
Gentamicin	(1) Additive	(0.314) Synergy	(1.25) Indifference

Note: ND denotes not determined and (number) denotes the ΣFIC index value.

**Table 5:** Interactions of 3β-acetylursolic acid and the different antibiotics

	***L. grayi***	***L. monocytogenes***	***L. ivanovii***
Ampicillin	ND	(1.11) Indifference	(1.11) Indifference
Penicillin G	ND	(1) Additive	(0.498) Synergy
Neomycin	(0.264) Synergy	(1.06) Indifference	(0.276) Synergy
Gentamicin	(0.25) Synergy	(0.156) Synergy	(1.25) Indifference

Note: ND denotes not determined and (number) denotes the ΣFIC index value.

### Membrane damage activity of the triterpenes

The triterpenes showed low levels of cytosolic lactate dehydrogenase release as shown in Table 6. The overall highest cytosolic lactate dehydrogenase released was 18.8% against *L. monocytogenes* by 3β-hydroxylanosta-9,24-dien-21-oic acid and the lowest being 0.02% against *L. ivanovii* by 3β-hydroxylanosta-9,24-dien-21-oic acid .

**Table 6:** **Membrane damaging activity (% cytosolic lactate dehydrogenase released) of the triterpenes against the** ***Listeria*** **bacteria**

**Test Triterpene**	***L. grayi***	***L. monocytogenes***	***L. ivanovii***
3β-hydroxylanosta-9,24-dien-21-oic acid	0.758	18.8	0.020
methyl-3β-hydroxylanosta-9,24-dien-21-oate	0.808	1.42	0.668
3β-acetylursolic acid	0.334	0.090	2

## Discussion

Triterpenes especially those isolated from medicinal plants have a high potential of use as pharmacological agents [[11]]. Plant compounds are usually classified as being antimicrobial based on MIC ranges of 100 to 1000 μg/ml while, those of typical antibiotics produced by bacteria or fungi are acceptable antimicrobials at much lower ranges of MICs between 0.01 and 10 μg/ml [[25]]. The triterpenes used in this study showed appreciable antibacterial activities against the three *Listeria* species although the 3β-acetylursolic acid had MICs outside the range acceptable for plant compounds. The triterpenes even had activities against *L. grayi* which was resistant towards the first line antibiotics of choice ampicillin and penicillin G. *L. grayi* is not normally a pathogenic species however, Salimnia *et al*. [[26]] reported a case of *L. grayi* bacteremia in a stem cell transplant recipient, showing the potential pathogenicity of this species as well, such that reports of its resistance to the penicillins are worrisome.

Combined therapy in listeriosis treatment is a well-accepted concept as shown by the different treatment options against listeriosis mentioned previously [[7],[8]]. In this connection, interactions between the triterpenes and the antibiotics in the penicillins (penicillin G and ampicillin) and aminoglycosides (gentamicin and neomycin) groups were tested in this study. Generally penicillin G had the most favourable interactions with the triterpenes in comparison to all the other antibiotics as it had more synergistic interactions with the triterpenes, while ampicillin had the least favourable interactions. The contrasting results observed between how the penicillins interacted with the same triterpenes against the same organism can be attributed to the differences in their chemical structures. Penicillins consist of a thiazolidine ring connected to a beta-lactam ring attached to a side chain. Ampicillin has an amino group on the benzyl side chain and this differentiates it from penicillin G of which the side chain determines most pharmacologic characteristics of each penicillin [[27]].

Generally 3β-hydroxylanosta-9,24-dien-21-oic acid was the triterpene that exhibited mainly synergistic and additive interactions with all the antibiotics against all the test *Listeria*. New findings of anti-*Listeria* agents that are non-toxic and possess synergistic interactions with conventional antibiotics against *Listeria* bacteria are significant as the current combinations involving gentamicin have nephrotoxicity risks [[28],[29]], while those involving sulfamethoxazole-trimethoprim have potential risks of causing kernicterus to the foetus and folic acid metabolism disturbances in pregnant women [[7]]. The triterpenes used in this study have however, been found to be non-toxic (with IC_50_ values that were higher than 300 μg/ml) against some tested human cell lines [[15],[17],[18]]. Compounds are considered significantly toxic when they have an IC_50_ value of less than 30 μg/ml [[17],[30]].

The MBC determination assay showed all the triterpenes to be generally bacteriostatic against the *Listeria* bacteria, however, in a bid to understand more on how the triterpenes effect their anti-*Listeria* activities *in-vitro*, the cytosolic lactate dehydrogenase test was carried out to determine if membrane damage is their mode of action. The results however, indicated that membrane damage is not the main mode of action of the triterpenes. This was evidenced by the low percentage of cytosolic lactate dehydrogenase released from the cells treated with the triterpenes (Table 6). Essential oils on the other hand which are typically a mixture of terpenes and/or terpenoids are postulated to exhibit anti-bacterial activity by disrupting the permeability barrier of microbial membrane structures [[31]], with some studies even showing the membrane disrupting activities of some plants essential oils against both Gram negative and Gram positive bacteria [[24],[31]].

The findings of this study however, have shown membrane damage by the triterpenes to be very minimal, such that it can be hypothesised that the triterpenes act on multiple target sites and their major mode of action may include inhibition of macromolecular synthesis such as DNA or RNA synthesis. The mechanism of action of some triterpenoids against Gram positive bacteria have also been shown to be a combination of plasma membrane disruption and inhibition of macromolecular synthesis such as RNA synthesis [[32]]. A study by Cristani *et al*. [[33]] also showed that the antimicrobial activities of some terpenes to be probably a resultant of partial agitation of the lipidic fraction of the plasma membrane in addition to the terpenes interacting with intracellular sites which are critical for bacterial growth [[33]].

The resultant synergistic results observed in this study can hypothetically be a combination of the protein synthesis inhibition properties of the aminoglycosides [[34]] or the cell wall inhibition properties of the penicillins (especially penicillin G in this study) [[35]] and the macromolecular synthesis inhibition properties of the triterpenes. In addition to the findings of this study, some previous studies have shown the methyl-3β-hydroxylanosta-9,24-dien-21-oate and 3β-hydroxylanosta-9,24-dien-21-oic acid to possess anti-inflammatory, anti-hyperlipidemic, anti-coagulant activities [[15],[18]], while the 3β-acetylursolic acid has been shown to possess anti-*plasmodial* activities [[17]].The various bioactivities of the triterpenes together show their multiple beneficial health effects potentials, such that their use in listeriosis treatment especially in the immune-compromised is highly likely to be advantageous.

## Conclusion

The three triterpenes tested possessed varying levels of anti-*Listeria* activities and also some synergistic interactions with conventional listeriosis treatment antibiotics *in-vitro* showing their potential in alternative listeriosis treatment. A follow up of the study with *in-vivo* tests would be highly recommended to ascertain if the observed *in-vitro* results align with the *in-vivo* results and also to determine if the synergistic interactions observed also result in bactericidal action.

## Competing interests

The authors declare that they have no competing interests.

## Authors’ contributions

DP wrote the manuscript and carried out the experimental studies. RM and MS supplied the triterpenes. All the authors participated in the design of the study and the critical revision of the manuscript and data content. All authors read and approved the final manuscript.
